# Can the outcome of post partum cardiomyopathy be predicted using late gadolinium enhancement CMR?

**DOI:** 10.1186/1532-429X-15-S1-P258

**Published:** 2013-01-30

**Authors:** Isabelle Roussin, Chirine Parsai, Sanjay K Prasad, Dudley Pennell

**Affiliations:** 1 Royal Brompton Hospital, London, UK

## Background

Post partum cardiomyopathy (PPCM) can occur between the last trimester of pregnancy or within 5 months post partum, in the absence of any history of heart disease. Although rare, it may become unresponsive to medical treatment and can in such cases, require mechanical support or even a heart transplant. Predicting its evolution is unfortunately not possible.

Late gadolinium enhancement CMR (LGE) can identify myocardial fibrosis and has shown its prognostic significance in DCM. However, the role of LGE in PPCM as a predictor of outcome has not been reported.

The purpose of this study was to assess whether LGE imaging provides prognostic information in the outcome in post partum cardiomyopathy.

## Methods

From 2003 to 2012, 36 women (mean age 34±10 years) diagnosed as PPCM underwent standard CMR with LGE following Gd-DTPA injection at the time of PPCM suspicion and after a mean follow-up period of 33 ±20 months for 21 of them (58%).

## Results

At the time of diagnosis: All patients showed increased left ventricular (LV) volumes (mean LVEDV: 201±49ml, mean LVESV: 101±50 ml), 17 patients showing reduced LVEF (mean LVEF 47.8% ±14%) and 15 patients had additionally dilated RV (mean RVEDV: 151.8±40.1ml, mean RVESV: 68.9±28.1ml). Only 7 patients displayed additionally reduced right ventricular (RV) function (mean RVEF 54.85±13.5%).

CMR data analysis from all patients in Table 1.

**Table 1:** 

	LVEDV (ml)	LVESV (ml)	LVEF (%)	RVEDV (ml)	RVESV (ml)
Baseline	201±49	108±50	47±14	151±40	69±28
Follow-up	177±39	78±26	56±8	153±40	66±20
p-value	0.018	0.003	0.005	0.5	0.8

Initial study showed mid wall LGE in 6 patients (30%) indicating myocardial fibrosis. Among them, one patient had a follow-up scan displaying persistence of myocardial enhancement but improvement in LV volumes and ejection fraction (EF). Four patients out of 6 had dilated LV with reduced LVEF; one showed increased volumes but normal EF and 1 had normal volumes with impaired EF. The RV appeared dilated with impaired EF in 3 patients at baseline (3/6 pts) without improvement on the follow up scan.

All the remaining patients without LGE (30/36 pts) had initially a dilated LV, (mean LVEDV: 195±40 ml, mean LVESV: 99.8± 38ml, mean LVEF 50%± 12) with associated reduced LVEF in 13 pts. Among them, 3 had a complete normalization of their LV volumes and 4 patients had normal LVEF at follow up. RV volumes and RVEF did not change significantly at follow up in this subgroup.

None of the patients showed active myocardial inflammation or oedema on STIR images.

## Conclusions

In our cohort, the presence or absence of myocardial late gadolinium enhancement was not predictive of LV functional recovery in PPCM suggesting that mechanisms other than myocardial fibrosis are involved in the genesis of ventricular dysfunction. None of our patients displayed myocardial edema. However, follow-up results are still awaited.

## Funding

None

